# Multifunctional metallic nanocomposite for overcoming the strength–ductility trade-off

**DOI:** 10.1038/s41598-023-50967-8

**Published:** 2024-01-10

**Authors:** Asheesh R. Lanba, Reginald F. Hamilton, Adrien N. Melanson, Emma S. Perry, Richard F. Gordon

**Affiliations:** 1 Department of Engineering Science and Mechanics, Pennsylvania State University, 212 Earth-Engineering Sciences Bldg., University Park, PA 16802-6812 USA; 2 Department of Engineering, University of Southern Maine, 37 College Ave., Gorham, ME 04038 USA; 3 Composites Engineering Research Laboratory (CERL), University of Southern Maine, 96 Falmouth Street, Portland, ME 04103 USA; 4 Electron Microscopy Laboratory, University of Maine, 23 Flagstaff Road, Orono, ME 04469 USA; 5 Medical Metals, LLC, Ridgefield, CT 06877 USA

**Keywords:** Composites, Metals and alloys

## Abstract

The actualization of high strength and ductility in alloys, in addition to providing strong, formable materials, can lead to reduced weights in practical applications. However, increasing strength typically comes at the cost of lowering the ductility and vice-versa, referred to as the strength–ductility trade-off. In this work, we investigate the thermo-mechanical response of a 3-element multifunctional NiTi–Nb nanocomposite material that overcomes this trade-off, as it exhibits a high strength of 980 MPa and an ultrahigh ductility of 58% at fracture. The remarkable properties are attributed to the underlying microstructure of Nb nanofibers dispersed in an NiTi matrix. Deformation is accommodated via the shape memory transformation of the active NiTi matrix in concert with elastoplastic deformation of Nb nanofibers embedded within the matrix. Consequently, the material exhibits multifunctionality and recovers deformation during heating via the reversion of the stress-induced martensitic transformation in the NiTi matrix. The high strength and high ductility of this 3-element nanocomposite material puts it amongst the best performing high-entropy alloys (HEAs) that are typically made up of five or more elements.

## Introduction

Despite alloys being at the forefront of mankind’s progress for thousands of years and the myriad of applications from infrastructure to medical tools to transport, there is a burgeoning demand for novel high strength and high ductility materials. Such materials can facilitate developing newer and sustainable applications by reducing amounts of alloy materials required which can enhance energy efficiencies^1^. Traditional mechanisms for increasing strength typically come at the cost of lowering ductility and vice-versa, which is referred to as the strength–ductility trade-off^1–3^. Recent work has focused on mixing five or more elements to create high-entropy alloys (HEAs) that overcome this trade-off^1,4,5^, but these alloys are not easy to manufacture when compared to ternary alloys and require many expensive starting materials along with specialized processing techniques.

**Figure 1:**
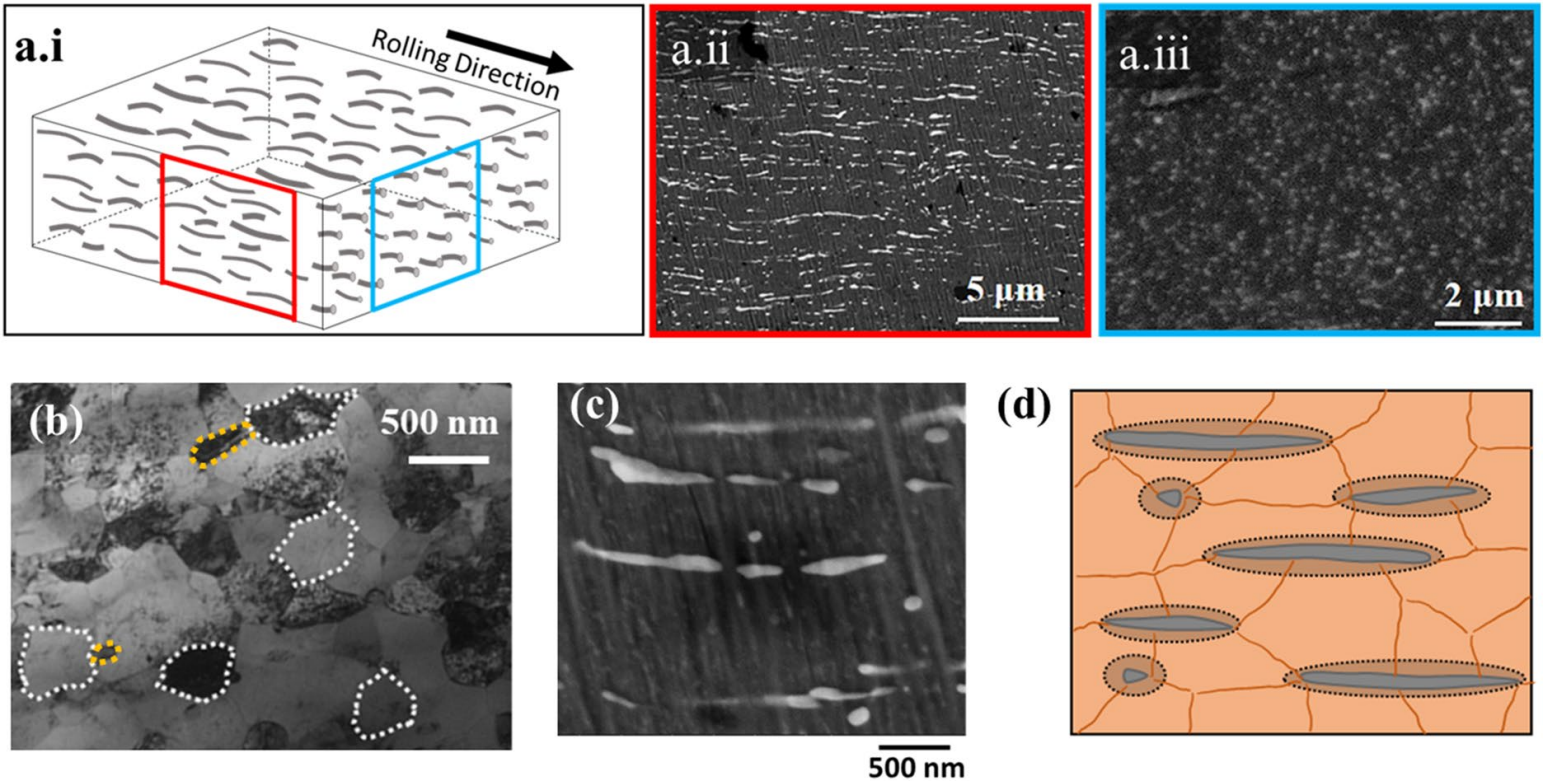
The 3D schematic of the representative volume element (RVE) is shown in (**a.i**), which is reconstructed from the rolling direction microstructure in (**a.ii**) and the transverse direction in (**a.iii**). These SEM micrographs in (**a.i,a.ii**) show that the microstructure consists of white Nb nanofibers embedded in the darker NiTi matrix. The higher magnification image in (**b**) shows the grain structure of the NiTi matrix (NiTi grains highlighted in white dashes) and Nb nanofibers (highlighted in yellow dashes). The image in (**c**) at a similar magnification shows the geometry of individual white Nb nanofibers. The 2D schematic in (**d**) shows the Nb nanofibers in grey along with the region of plastic deformation highlighted via the darker dotted regions that form during deformation embedded in the orange NiTi matrix with the grain boundaries highlighted. The micrographs in (**a,c**) were obtained via SEM, and the micrograph in (**b**) was obtained via TEM.

The microstructural morphology of our NiTi–Nb nanocomposite in Fig. 1a consists of Nb nanofibers dispersed in an NiTi matrix. Similar nanostructured alloys have leveraged the ability to elastically deform up to 4–7% strain under stresses larger than the yield strength (a significant fraction of their ideal strength) of bulk materials when deformed non-hydrostatically (for e.g., in tension)^6^. Thus, we envisage NiTi-Nb processing leveraging micro- and nano-composite design solutions^7^. Previous work has shown an example of this microstructure; continuous Nb nanowires coexist within an active NiTi matrix that undergoes a phase change due to the underling martensitic transformation (MT) that takes place via twinning and subsequent detwinning as stress/strain levels increase^8^. These embedded nanowires have large elastic strains like those of free-standing nanowires (~ 3.5%) that are higher than the elastic strain embedded in conventional metal matrices that deform by dislocation slip (~ 1.5%)^8^. Thus, using these nanostructured materials allows for elastic and inelastic deformation matching to control properties such as the martensitic phase transformation^6^.

Novel nanocomposite materials have used this concept of lattice strain matching between uniform lattice distortion of the martensitic phase transformation in the matrix material and uniform ultra-large elastic/plastic strains of Nb nanoscale microconstituents^6,8–14^. Hao et. al showed that in such materials, the large elastic strains of Nb nanowires couple with the superelastic/pseudoelastic shape memory strain in the active NiTi matrix^10^. This coupling resulted in remarkable mechanical behavior and properties; recoverable strain that exceeds 6%, a relatively low Young’s Modulus that is nearly 30 GPa, and a ultrahigh yield strength of 1.65 GPa^10^. The length of these Nb nanowires ranged between 1 and 100 µm with an aspect ratio exceeding 100^10^. A synergistic effect of strain matching produced inhomogeneous elastic deformation in Nb nanowires, with nearly 8% strain induced in the Nb nanowire regions near the NiTi undergoing the stress-induced MT, and much lower strains in regions near untransformed austenitic NiTi matrix^14^. Our NiTi–Nb material is comprised of discontinuous Nb nanofibers embedded in an NiTi matrix (as shown in the represented volume element in Fig. 1a.i that is recreated using the SEM images in the rolling and transverse directions in Fig. 1a.ii, a.iii respectively), with mean lengths of less than 500 nm and a lower aspect ratio (< 10) that results in a material with both high strength and ductility, and has the ability to recover imparted deformation via the shape memory effect (SME).

## Results

**Figure 2:**
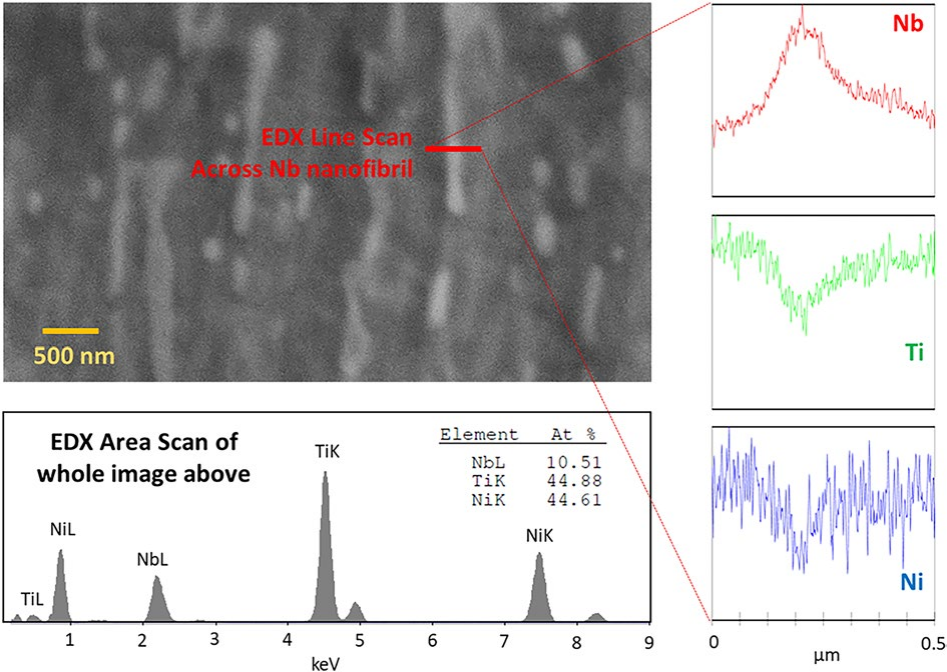
SEM-energy dispersive X-ray analysis (EDX) line going across a white Nb nanofibril clearly shows that is almost pure Nb, with the Nb concentration increasing and the Ni–Ti concentrations decreasing across the line. The whole area EDX results show that the at% distribution of Nb, Ti and Ni for the micrograph are close to the supplier’s concentration, thus validating EDX results.

The microstructure of this anisotropic NiTi-Nb nanocomposite consists of discontinuous Nb nanofibers dispersed in an NiTi matrix, as shown in Fig. 1a. The SEM images are taken along the processing/rolling direction (Fig. 1a.ii) and perpendicular to it (Fig. 1a.iii) that were used to quantify the sizes of the Nb nanofibers. The mean length of the fibers is 452 nm, with a standard deviation of 273 nm and a range of 5 to 940 nm. The mean width of the fibers is 190 nm with a standard deviation of 105 nm and a range of 2 nm to 375 nm. Figure 1b is a TEM image showing the grain structure of the NiTi matrix (a few grains are highlighted in white dashes, and some Nb nanofibers in yellow dashes), and the representative grains are highlighted with sizes approaching 300 nm, with the smallest grains close to 100 nm and the larger grains approaching 400 nm. The light and dark contrast in the grains is related to the amount of electrons being transmitted through the structure, and is related to the different orientations of the grains. Many of the grains appear lighter potentially due to preferential orientation in the rolling direction. The higher magnification SEM image in Fig. 1c shows discontinuous Nb nanofibers are oriented in the rolling direction and coexist with nano-spheroids. An energy dispersive X-ray analysis (EDX) line scan in the high magnification micrograph in Fig. 2 shows that the nanofibers have a much larger Nb concentration and much lower Ni/Ti concentration than the surrounding NiTi matrix.

**Figure 3:**
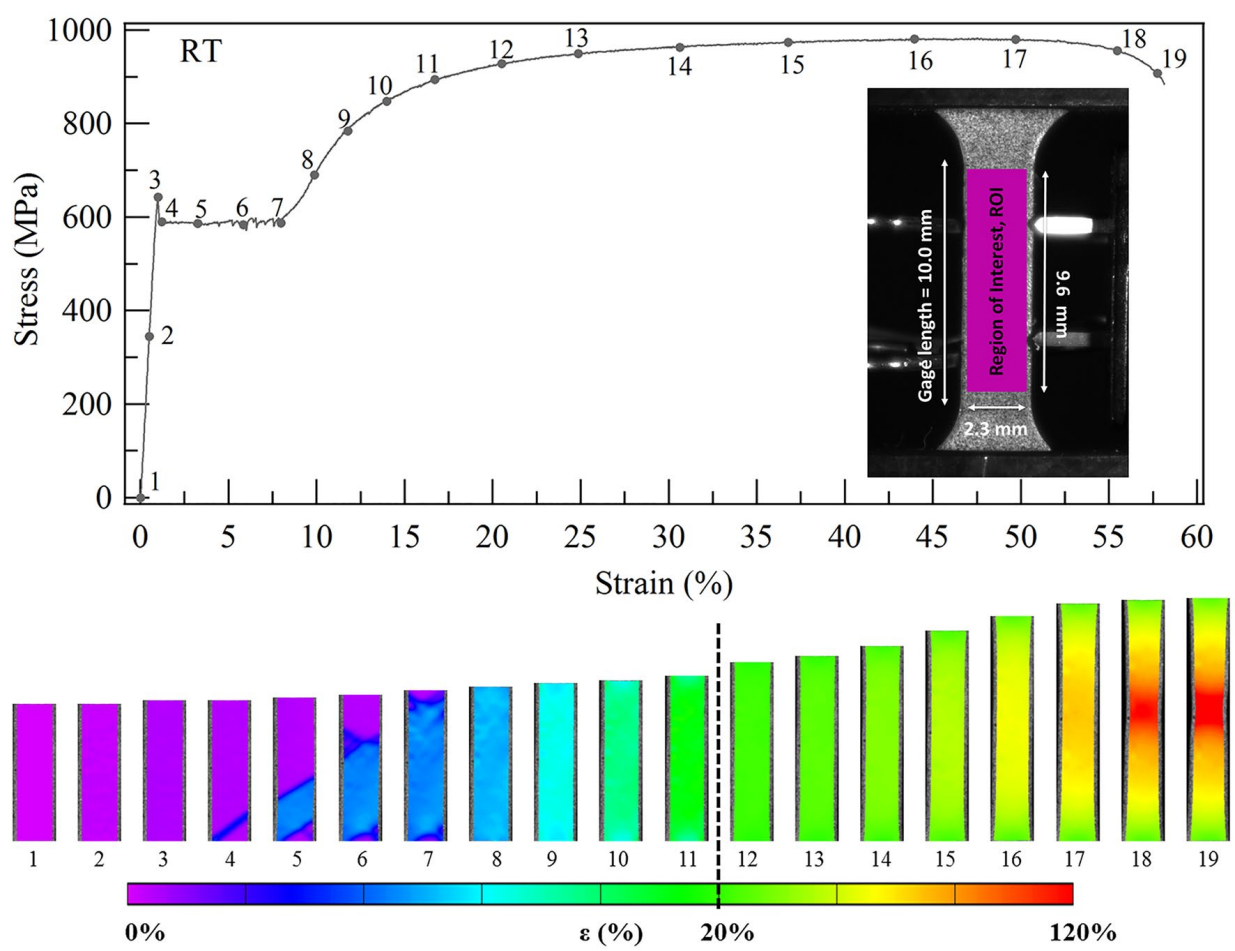
The room-temperature tensile stress–strain response of the NiTi-Nb nanocomposite model highlights the material’s outstanding strength and ductility. The accompanying DIC images show the morphology of localized strains in the loading direction at discrete points along the stress-stress response. The active region of 20% is delineated on the strain scale for the DIC images. The inset figure shows the sample geometry and location of region of interest for the DIC images.

The uniaxial room temperature tensile stress–strain (σ–ε) plot in Fig. 3 shows the mechanical response to failure. The NiTi–Nb nanocomposite possesses striking mechanical properties; a low Young’s Modulus of 64.3 GPa, very high ultimate tensile strength of 980 MPa, a large ductility with a strain at fracture of 58%, and a high tensile toughness of 514 MJ/m^3^. Also shown are accompanying full-field strain maps from DIC analysis of axial deformation measurements within the specimen gage section, which is defined as the region of interest (ROI). When linear-elastic response ends, the stress drops, and the stress-induced martensite (SIM) volume fraction grows causing the σ-ε response to plateau at a transformation stress of approximately 590 MPa. The SIM produces a localized strain contour band in DIC image 4 in Fig. 3, analogous to a high-strain Lüders bands. As the band grows across the gage length in images 4–8, the maximum localized strains saturate around 9%. Beyond the plateau, the elastic/plastic deformation of the detwinned martensite takes place^15^. Note that the contours change color homogeneously in images 8–10, which is typical for elastic deformation. Images 10–15 are captured during a second linear-to-nonlinear transition. It is well known that martensite plastically deforms, which is expected to produce strain localization^15^. Apparently, corresponding local strains are undetectable using our DIC measurement length scales. Images 16–19 correspond to the necking response, where stress begins to decrease with increasing strain. A localized region of very high strain appears in the center of the specimen where the strains approach 120%, and the specimen eventually fractures at this location.

**Figure 4:**
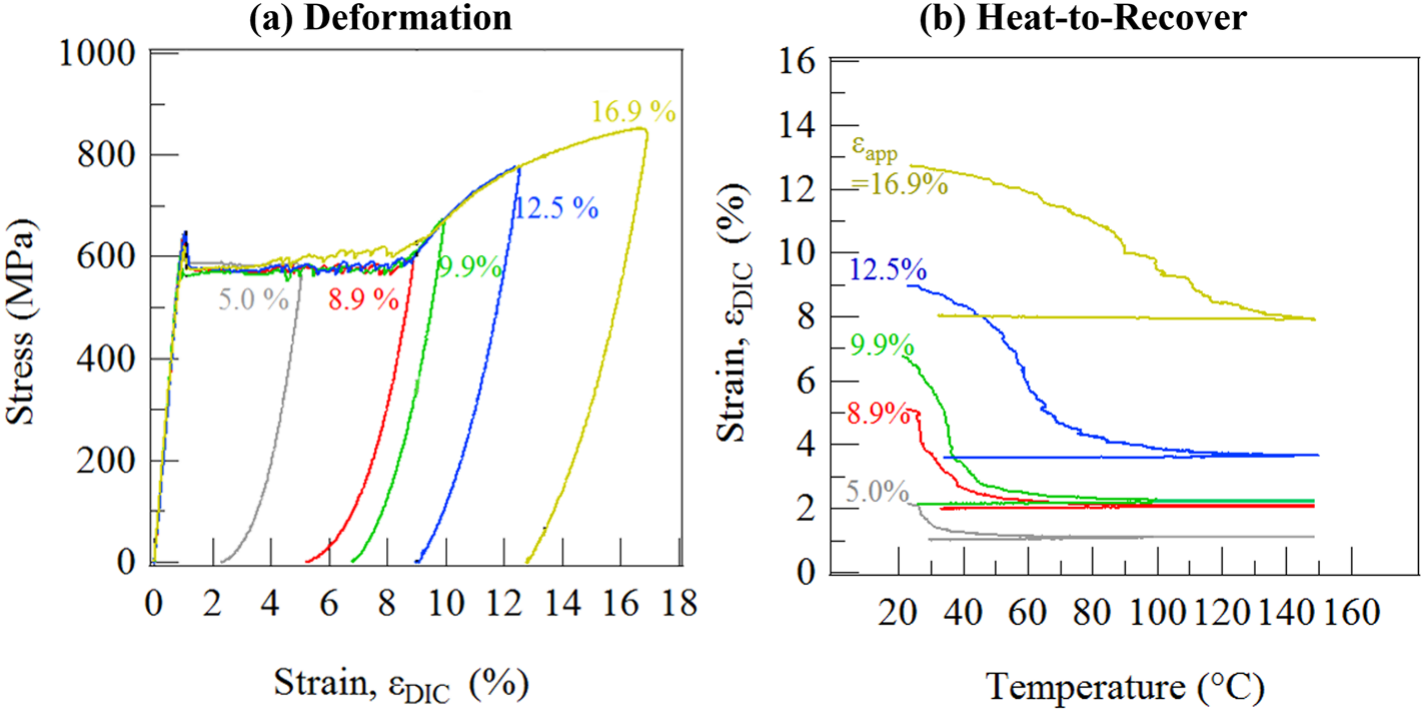
The active property of shape memory recovery of these NiT–Nb nanocomposite material is demonstrated in (**b**) after deforming different samples to different levels of increasing strains in (**a**). All specimens were heated to 150 °C from room temperature and then allowed to cool back down to room temperature.

The shape memory recovery behavior that shows the material’s active behavior is shown in Fig. 4. In Fig. 4a, strain recovery produces an initial linear-elastic unloading σ-ε response. A distinct non-linear response follows the linear-elastic unloading segment for 5.0% that is indistinguishable at 16.9%. The non-linearity is attributed to partial superelastic shape memory recovery as SIM reverts to the austenite parent structure. After unloading from the active region (subjected to < 20% strain), samples were heated, and strain recovery takes place due to the reversion of SIM that has been stabilized in the NiTi matrix by loading deformation. Stable SIM remaining after unloading is remarkable. Superelastic recovery would be expected at room temperature as the material is in its austenitic state and fulfills the criteria for pseudoelasticity^16–18^. The stabilized SIM can be considered as “atypical” with respect to the primary SIM that formed at the stress-plateau. As the pre-strain levels increase, higher temperatures are required for complete reversion of the atypical martensite via the shape memory effect (SME). Only a fraction of the applied deformation/strain was recovered during unloading and heating, during linear-elastic unloading, SE recovery, and SME recovery, and permanent residual strain always remained. Deforming to 10% results in the largest recovered strain of 5% and the highest relative recovery (ratio of recovered strain via heating to the permanent deformation that remained).

## Discussion

**Figure 5:**
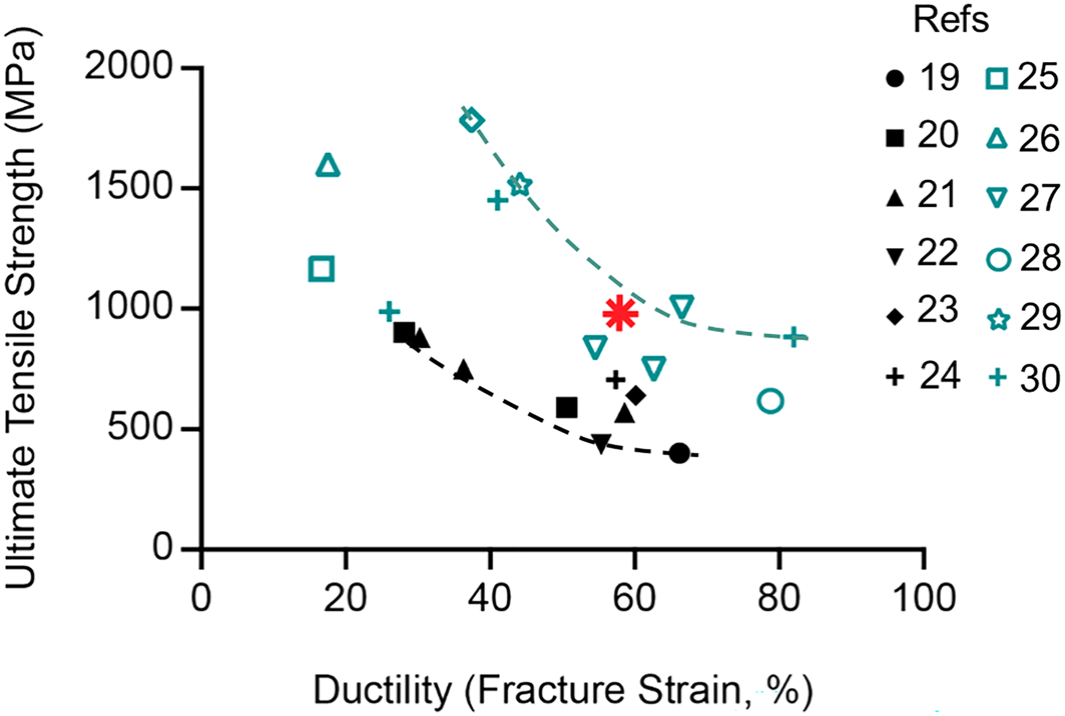
This plot shows the relative location of the ultimate tensile strength and ductility of the active NiTi–Nb nanocomposite material and those of high entropy alloys and stainless steels from literature. The green points represent the best-performing high entropy alloys (HEAs). The black points represent the best-performing 316L stainless steels. Our NiTi-Nb nanocomposite material is the red star. This red star lies amongst the best performing high-entropy alloys. It is also stronger than all the stainless steels and is on the higher ductility side of the steels as well. All the green and black data points are from references^19–30^.

Figure 5 shows that our multifunctional NiTi-Nb metallic nanocomposite outperforms all the best performing 316L stainless steels^19–24^. The processing needed to produce these high strength and high ductility steels are solution nitriding^19^, ultrashort annealing at different temperatures to produce ultrafine grains^20^, multi-step dynamic compression to produce nanotwinned grains^21^, additively manufactured hierarchical microstructure^23^, and additively manufactured textured microstructure^24^.

Incredibly, the ultimate tensile strength and ductility of this nanocomposite material is among the best performing high-entropy alloys, as shown in Fig. 5^25–30^. Gao et al. studied an AlCoCrFeNi_2.1_ eutectic HEA produced having modulated lamellar structure^25^. Li et al. investigated an Al_0.3_CoCrFeNi HEA that possessed nanosized particles reinforcing an austenitic matrix^26^. FeMnCoCr and CoCrFeMnNi HEAs showed high strength and high ductility at a cryogenic temperature of 77 K^27,30^. Zhang et al. analyzed a dual-phase FeCoCrNiMn HEA, with one phase contribution to high strength and the other to high ductility^28^. Jo et al. examined an FeCMnSiMoV HEA reinforced by segregated Mn bands^29^.

Our nanocomposite alloy is also multifunctional; it has an active region when deformed to strains less than 20%, as shown in Fig. 4. This partial recovery is due to the shape memory effect in the NiTi matrix. The austenitic NiTi in the matrix transforms to stress-induced martensite (SIM) that is stable after unloading. This SIM is transformed back to austenite upon heating and the material and recovers residual strain due to the shape memory effect (SME)^31^. However, there is only partial recovery as the stress-induced transformation in the matrix is accompanied by the permanent plastic deformation of the Nb nano fibers. These nanofibers will not recover deformation upon heating, and likely affect surrounding stress-induced martensite to not recover as well (as shown schematically in Fig. 1d), resulting in permanent deformation not recovered via heating.

Plausible mechanisms for the high strength of the material are related to the microstructural features that act as obstacles for dislocation motion^32^. For our NiTi-Nb nanocomposite material, these include (i) high grain boundary densities attributed to the nanosized grains and (ii) the presence of Nb nanofibers. Additionally, the strain-induced martensitic transformations are facilitated by much higher stresses compared to SIM. Rather than slip dislocations coalescing into a crack that grows until ductile fracture occurs, the strain-induced martensite curtails macro-scale necking. We postulate that the SIM and strain-induced martensite become stable so that only a fraction of martensite reverts via superelastic recovery. For SMAs, stabilization typically refers to martensite that becomes trapped and requires heating to higher temperatures above the characteristic phase transformation temperature^33^. Further recovery takes place during heating via SME, albeit a fraction of the applied strain is recoverable.

Possible mechanisms for the high ductility of the materials are related to microstructural features that can accommodate large deformations prior to fracture. The Nb nanofibers can elongate extensively, withstanding the highest levels of plastic deformation^10^. Our microstructural analysis shows that the Nb nanofibers possess a length to width aspect ratio that ranges between 0.6 and 8.5. These nanofibers can further plastically deform to a much larger aspect ratio, closer to the higher aspect ratios > 100 achieved by Nb nanowires in the NiTi-Nb nanocomposite material studied by Hao et.al.^10^. Additionally, the dotted boundaries encompassing fibers in Fig. 1d depict a localized region of active NiTi adjacent to the fibers that can inherit the plastic deformation once the externally applied stress/strain level reaches a critical level. In local regions near these grains and/or in the heavily plastically deformed regions adjacent to the Nb nanofibers, the active matrix deforms further as SIM undergoes subsequent transitions to strain-induced martensite^34^. As a result, remarkably high strain levels are achieved prior to and during necking deformation. Thus, the microstructural features of highly pliable Nb nanofibers, undeformed grains and an NiTi matrix that undergoes the stress-induced martensitic transformation results in this active nanocomposite material that overcomes the strength/ductility tradeoff.

## Methods

A Ni_47.7_Ti_43.5_Nb_8.8_ at.% alloy in rolled strip form (6 mm wide and 0.25 mm thick) was supplied by Medical Metals LLC. The material was prepared via multiple thickness reductions through cold rolling of an ingot made via vacuum induction melting, followed by annealing near recrystallization temperature (850 °C). Previous differential scanning calorimetry analysis did not reveal endothermic or exothermic peaks^18,35^. Consequently, the transformation temperatures were measured by applying a constant bias load (equivalent to 150 MPa stress) during thermal cycling: $M_{s} \, = \, - 64 \, ^\circ {\text{C}}$; $M_{f} = \, - 76 \, ^\circ {\text{C}}$; $A_{s} \, = \, - 30 \, ^\circ {\text{C}}$ and $A_{f} \, = \, - 7 \, ^\circ {\text{C}}$^18^. Scanning electron microscopy (SEM) and transmission electron microscopy (TEM) were carried out at room temperature. For SEM, specimens were polished via SiC paper with grit size decreasing from 180 to 1200 and finally polished using 0.02 μm colloidal silica. The imaging was performed in a Philips XL30 ESEM, while EDX was performed in a Zeiss NVision 40 SEM. The fibril sizes were determined from the rolling direction micrograph in Fig. 1a.ii. The image was analyzed in ImageJ software by thresholding out the nanofibers^36^. The length and the width of the nano-fibril was determined using the Feret’s diameter in the rolling and transverse direction respectively in the first micrograph in Fig. 1a.ii^37^. For TEM, specimens were mechanically polished to 10 μm thickness and made electron transparent using focused ion milling. The thinned specimen was attached to a molybdenum grid and ion milled from both sides in a Gatan Precision Ion Polishing System at a beam angle of 15° with an accelerating voltage of 3 kV. The imaging was performed in a Philips 420 transmission electron microscope operated at 120 kV using a single-tilt holder. The grain size was determined from Fig. 1b using the Mean Intercept Procedure per ASTM E112-12^38^.

The tensile testing was performed in an MTS 810 servo hydraulic load frame at room temperature, and this frame was equipped with a custom induction coil heating set-up. The temperature was measured via a thermocouple attached to the specimen. Tensile specimens with dog-bone geometry were electro-discharge machined from the rolled strip such that the gage length was 10 mm and gage width was 3 mm, as shown in the inset of Fig. 3. A single specimen was deformed up to one of five pre-strain levels (5.0, 8.9, 9.9, 12.5 and 16.9%) so that five virgin specimens were pre-strained to investigate OWSME recovery. The specimens were loaded in displacement control at a rate of 0.0028 mm/s, which corresponds to an average strain rate of 1.7 × 10^–4^ /s. After the strain during loading reached the desired level, the specimen was unloaded in force control at an equivalent stress rate of 1.2 MPa/s. The rates are in accordance with the ASTM E8 Standard Test Methods for Tension Testing of Metallic Alloys^39^. After unloading, the load was fixed at zero and the specimen was heated at a rate of 10–15 °C/min via induction.

The axial strain measurements for the stress–strain curves were determined using a virtual digital extensometer feature in the digital image correlation software, referred to as an inspect extensometer (IE). The general steps for implementation of DIC analysis are specimen preparation, machine vision set-up and image acquisition, and image correlation. The correlation theory has been explained in the works of Sutton et al.^40–42^. A speckle pattern was applied on the specimen surface using an IWATA Micron-CMB airbrush. A very thin and uniform white coating of Golden Airbrush Titanium White (#8380) paint was applied as the background for a black micron speckle pattern of Golden Airbrush Carbon Black (#8040) paint. In-situ images of the spray-painted specimen surface were captured using a Grasshopper GRAS-20S4M/C CCD camera (1600 × 1200 pixels). Digital images had a resolution of 58.7 pix/mm. Image capture was synchronized with load and displacement data acquisition using Vic-Snap system (Correlated Solutions, Inc.). DIC numerical analysis was carried out using Vic-2D^®^ software (Correlated Solutions, Inc.). The undeformed length of the IE is equivalent to the specimen gage length. Strain is calculated as the change in length divided by the undeformed length and is referred to as average/macroscale.

In-situ full-field deformation measurements during pre-straining and OWSME recovery were calculated using digital image correlation (DIC). For the analysis, a region of interest (ROI) is selected and divided into subsets. The ROI in this work spans the specimen gage length and it measures 2.4 × 9.4 mm^2^, as shown in the inset of Fig. 2. DIC analysis can be considered as measuring strain over the specimen surface using micron sized strain gages; the size is defined by the subset size and spacing^43^, and was 270 μm for this work.

## Data Availability

The datasets generated during and/or analyzed during the current study are available from the corresponding authors on reasonable request.
